# Learning from microarray interlaboratory studies: measures of precision for gene expression

**DOI:** 10.1186/1471-2164-10-153

**Published:** 2009-04-08

**Authors:** David L Duewer, Wendell D Jones, Laura H Reid, Marc Salit

**Affiliations:** 1 Analytical Chemistry Division, National Institute of Standards and Technology, Gaithersburg, Maryland 20899-8390, USA; 2 Expression Analysis, 4324 South Alston Avenue, Suite 101, Durham, North Carolina 27713, USA; 3 Biochemical Science Division, National Institute of Standards and Technology, Gaithersburg, Maryland 20899-8313, USA

## Abstract

**Background:**

The ability to demonstrate the reproducibility of gene expression microarray results is a critical consideration for the use of microarray technology in clinical applications. While studies have asserted that microarray data can be "highly reproducible" under given conditions, there is little ability to quantitatively compare amongst the various metrics and terminology used to characterize and express measurement performance. Use of standardized conceptual tools can greatly facilitate communication among the user, developer, and regulator stakeholders of the microarray community. While shaped by less highly multiplexed systems, measurement science (metrology) is devoted to establishing a coherent and internationally recognized vocabulary and quantitative practice for the characterization of measurement processes.

**Results:**

The two independent aspects of the metrological concept of "accuracy" are "trueness" (closeness of a measurement to an accepted reference value) and "precision" (the closeness of measurement results to each other). A carefully designed collaborative study enables estimation of a variety of gene expression measurement precision metrics: repeatability, several flavors of intermediate precision, and reproducibility. The three 2004 Expression Analysis Pilot Proficiency Test collaborative studies, each with 13 to 16 participants, provide triplicate microarray measurements on each of two reference RNA pools. Using and modestly extending the consensus ISO 5725 documentary standard, we evaluate the metrological precision figures of merit for individual microarray signal measurement, building from calculations appropriate to single measurement processes, such as technical replicate expression values for individual probes on a microarray, to the estimation and display of precision functions representing all of the probes in a given platform.

**Conclusion:**

With only modest extensions, the established metrological framework can be fruitfully used to characterize the measurement performance of microarray and other highly multiplexed systems. Precision functions, summarizing routine precision metrics estimated from appropriately repeated measurements of one or more reference materials as functions of signal level, are demonstrated and merit further development for characterizing measurement platforms, monitoring changes in measurement system performance, and comparing performance among laboratories or analysts.

## Background

The ability to demonstrate the reproducibility of gene expression microarray results is a critical element in their adoption for clinical applications. Several studies have asserted that microarray data can be "highly reproducible" if probe sequences are well-mapped to the genome and standard protocols are followed [1-3]. While largely focused on comparisons among measurement platforms, these and other studies have variously characterized many aspects of microarray performance. However, the microarray community has yet to adopt a standardized terminology and practice for characterizing performance that can facilitate clear communication among the user, developer, and regulator stakeholders.

The measurement science (metrology) community is devoted to establishing a philosophically coherent terminology and practice for characterizing and communicating measurement performance [4]. As the world's largest developer and publisher of international consensus standards, the non-governmental International Organization for Standardization (ISO) plays a critical role in disseminating this guidance [5]. The documentary standard ISO 5725-1 [6] details the basic concepts and estimation techniques for assessing metrological "accuracy" which is defined as a combination of two concepts, "trueness" and "precision." These concepts are formally defined in the Vocabulary of International Metrology (VIM) [7] base document and more cogently described in ISO 3534 [8]: trueness is the closeness of a measurement to an accepted reference value and precision is the closeness of measurement results to each other. While microarrays can generate vastly more data per sample than is typical of the processes that shaped the development of these documents, we believe that this pre-existing metrological framework can be extended to microarrays and other highly multiplexed measurement processes.

Properly designed collaborative studies are one of the very best ways of obtaining the information required to characterize some aspects of measurement performance [9]. The three "rounds" of the Expression Analysis Pilot Proficiency Test evaluated replicate samples of a pair of mixed-tissue RNA pools across multiple participants from June to December of 2004; these studies provide a wealth of information relevant to the estimation of several aspects of within-platform measurement precision and among-participant measurement concordance [10]. While the known relationships between the two RNA pools used in these studies also enable evaluation of several measures of trueness in *differential *expression [11], we here evaluate only the metrological concepts of precision as applied to the underlying *direct *measurements. These concepts provide a foundation for the development of objective expectations for the consistency of microarray results. We anticipate that this foundation will facilitate improving the comparability of microarray measurements over time and place and may lead to the development of new approaches and tools for objectively demonstrating the utility of measures of differential expression and ranked lists of differentially expressed genes.

## Results

The precision of a defined measurement process can be characterized using three nested metrics: "repeatability," "intermediate precision," and "reproducibility." These measures of precision are defined in terms of the conditions that apply when the measurements are obtained, including: operator, equipment, calibrations, environmental conditions, and the period of time between measurements. Repeatability is defined as the precision of independent measurements when all conditions are assumed constant and thus do not contribute variability (eg, single participants in a given round). Reproducibility is defined as the precision observed when all conditions are permitted to vary within allowable tolerances and thus to contribute variability (eg, all participants in at least one round). Intermediate precision is a special case, where some specified factors are held constant and others are varied (eg, a single participant in two or more rounds). A variety of analysis of variance approaches are suitable for dissecting such data. ISO 5725-2 details the standard approach used in measurement science to characterize measurement precision for a specific measurement process from the results for a single material in a single study [9,12].

### Classical precision metrics

In the context of the microarray platform measurements, the signal from each probeset of the array is a single measurement process. The ISO 5725-2 calculations for a single process in a single study are described in Methods, *Classical Precision Metrics for Single Studies*. Methods, *Classical Precision Metrics for Multiple Studies *extends the calculations to multiple studies that use nominally identical samples. The design elements of the Expression Analysis Pilot Proficiency Test studies that enable the use of these calculations are described in Methods, *Study Design*.

While tedious, none of the classical precision metrics are particularly complex or difficult to calculate. However, keeping track of the nomenclature and symbols used for the various metrics can be challenging. In quick summary: let *x*_*ijk *_represent the *k*^*th *^replicate measurement of a given probeset reported by the *j*^*th *^participant in the *i*^*th *^round. The usual mean, , and standard deviation, *s*(*x*_*ij*_), of the replicates estimate the value and technical variation for that probeset for the particular participant at one point in time. The value  and technical variation characteristic of the microarray platform itself are estimated by combining the  and *s*(*x*_*ij*_) values over all participants; the metrological term for this expected technical variation is *repeatability precision *and is represented as *s*_*ri*_. The variation among the different  estimates the *between-participant precision *and is represented as *s*_L*i*_. In combination, the two sources of variation estimate the *reproducibility precision *which is represented as *s*_*Ri*_. These single-round estimates can be regarded as characterizing the performance of the given probeset over the (typically short) duration of the study.

When nominally identical samples are analyzed in multiple studies, the resulting multiple  and *s*(*x*_*ij*_) values can be used to estimate the participant-specific expected value, , repeatability, *s*_*rj*_, and *among-round precision*, *s*_W*j*_; the combination of these two sources of variation estimate the *intermediate precision over time *for the participant, *s*_I(T)*j *_[13]. Combining the single-round, single-participant estimates over all participants can also estimate the long-term expected value, , repeatability, *s*_*r*_, between-laboratory precision, *s*_L_, and reproducibility, *s*_*R*_; these long-term estimates can be regarded as characterizing the intrinsic performance of the platform.

### Precision functions

The above classical precision metrics characterize performance for individual processes with multiple nominally identical samples of one material. Characterizing processes as a function of signal level can usefully identify the performance expected for "typical" samples [14]. For measurement methods involving one to a few tens of different measurement processes, this can be accomplished with interlaboratory studies involving a relatively small series of samples of similar matrix composition but with varying levels of the analytes of interest. If the many (thousands to millions) of signals typical of microarrays have very different measurement characteristics, then little simplification to this classical approach is possible. However, estimation of aggregate precision functions from analyses of a single material becomes feasible if the majority of the measurement processes share similar performance characteristics. The expected performance of a "typical" probeset with a "typical" sample can be established by compositing the multitudinous individual estimates for one or a few samples.

The Expression Analysis Pilot studies provide results for 31054 probesets in each of two mixed-tissue pools. In the following, we characterize the various precision metrics as functions of signal level from the 62108 unique combinations of probeset and material.

#### Discrete Precision Functions

**Figure 1:**
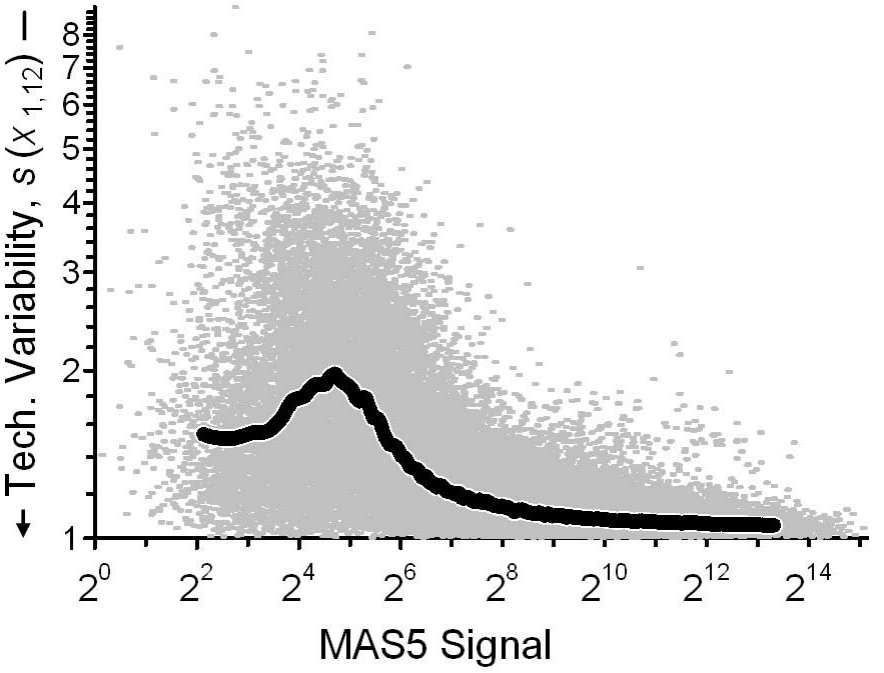
**Within-participant repeatability as a function of signal intensity**. Each flyspeck denotes the average signal intensity and repeatability, {*x*_1,12_,*s*(*x*_1,12_)}, for one of the 62108 sets of measurements made by participant 12 in Round 1. The thick line represents the precision function defined from the median of successive 1/100 binnings of these estimates.

Figure 1 illustrates a simple, empirical approach to evaluating relationships between signal level and precision metrics, where the multitude of individual {signal level, precision} values are reduced to a relative handful of expected values [15,16]. Each datum represents the average signal and standard deviation of the triplicate measurements for one probeset for one of the two mixed-tissue pools reported by participant 12 in Round 1, {,*s*(*x*_1,12_)}. The line displays the expected value of these estimates, {<>,<*s*(*x*_1,12_)>}, calculated as the medians of 100 equally sized groups of the {,*s*(*x*_1,12_)} after ordering on . While many summarization approaches could be used, this binning approach has the benefit of relative familiarity and computational simplicity.

**Figure 2:**
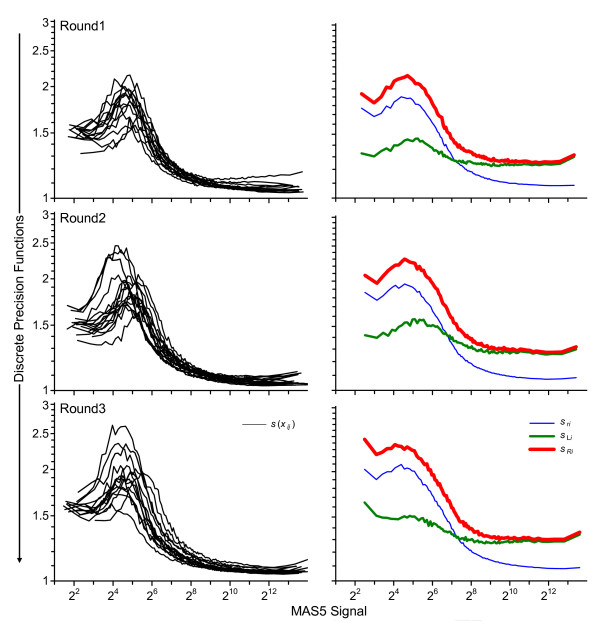
**Within-round discrete precision functions**. These panels present the single-round discrete precision functions for the three rounds. The panels to the left display participant-average signal intensities and repeatabilities, {<>,<*s*(*x*_*ij*_)>} for every participant in the round. The panels to the right display round-average intensity and repeatability, {<>,<*s*_*ri*_>}, between-participant precision, {<>,<*s*_*Li*_>}, and reproducibility, {<>,<*s*_*ri*_>}. In all cases the functions are estimated as the medians of successive 1/100 binnings.

**Figure 3:**
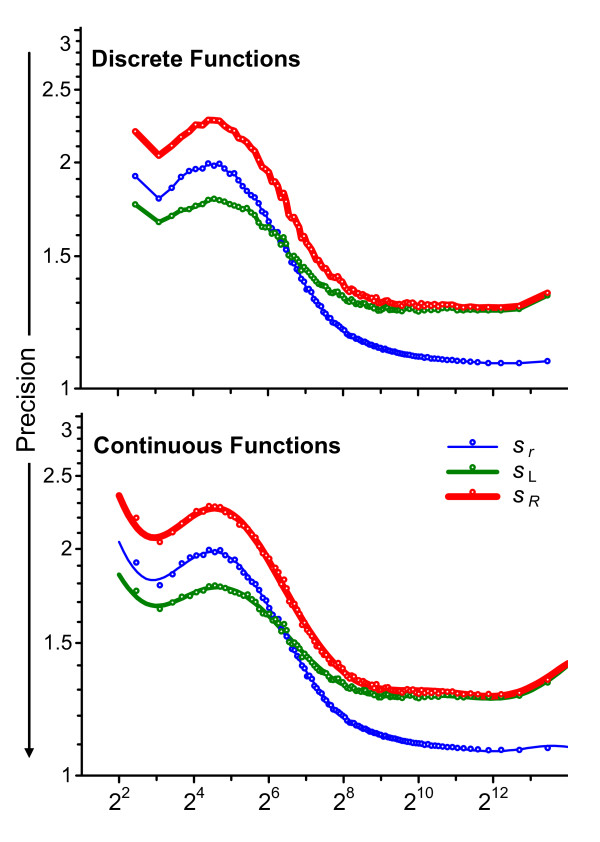
**Discrete and continuous precision functions characteristic of the method**. The upper panel displays the discrete between-round repeatability {<>,<*s*_*r*_>} (thinnest line), between-participant precision {<>,<*s*_L_>}, and reproducibility {<>,<*s*_*R*_>} (thickest line) functions. The lower panel displays the same precisions as continuous functions, in the form of 10th-order polynomials. In both panels, the open circles denote the values that define the discrete functions.

Figure 2 displays all of the precision functions for the three rounds: the {<>,<*s*(*x*_*ij*_)>} standard deviation functions for the individual participants, the {<>,<*s*_*ri*_>} repeatabilities, the {<>,<*s*_L*i*_>} between-participant precisions, and the {<>,<*s*_*Ri*_>} reproducibilities. The upper panel of Figure 3 displays the {<>,<*s*_*r*_>} between-round repeatability, the {<>,<*s*_L_>} between-participant precision, and the {<>,<*s*_*R*_>} reproducibility functions. The only difference in construction of these discrete functions is use of the appropriate average signal,  or , rather than the participant average, , in the initial list creation step.

The pattern and magnitude of the various functions in all three rounds are very similar to each other, indicating that the precision characteristics of the method did not change much over the seven months between Round 1 and Round 3 or with the change in number and identity of the participants. This pattern of technical variability as a function of signal level (in order of lowest to highest signal level: smoothly increasing to a maximum, smoothly decreasing to a relative constant minimum until a small increase at the very highest signal levels) has been often observed with Affymetrix Microarray Suite 5 [17] (MAS5)-processed data [15,18,19]. This complex structure appears to be at least mostly an artifact of MAS5 processing rather than an intrinsic property of the microarray platform, since it is not observed with some other data processing approaches [15,16,20]. Regardless, variability estimated from short-term studies may underestimate the variability expected over longer periods: while <*s*_*r*_> for the higher signal levels is a multiplicative factor of 2^0.14 ^= 1.1 or about 10% relative, <*s*_*R*_> is a factor of 2^0.38 ^= 1.3 or about 30% relative. It is perhaps noteworthy that these lessons can only be learned through longitudinal study of measurements from a reasonably well defined population of participants.

#### Continuous precision functions

While discrete functions are efficient as graphical summaries, they are inconvenient for estimating quantitative values of an expected precision at specific signal levels. For use in further calculations, such as variance-stabilized normalization [21,22], it is desirable to represent the various precision estimates as continuous functions,  = *f*(*x*), where  is the estimated precision value, *x *is the signal level, and *f *is some function parameterized with a relatively small set of coefficients. While a simple four-parameter sigmoidal curve captures much of the structure for most of the discrete functions for the present data, the underlying model assumption of monotonic change as the signal rises from the detection-limit to saturation-limit is not fully adequate to describe MAS5 behavior. Rather than attempting to define and fit more complete theory-based models for particular datasets, platforms, or data processing systems, interpolative empirical functions can readily capture the observed structure. While comparatively crude and unsuitable for extrapolation, even simple polynomials of modestly high-order provide a reasonable qualitative description of the dependence as well as being easily implemented and interpreted. The lower panel of Figure 3 displays 10^th^-order polynomials parameterized to the discrete between-round precisions shown in the upper panel of Figure 3. At graphical resolution, very similar fits to the discrete functions are provided by polynomials of order 7 and above.

## Discussion

### Characterizing measurement systems

The between-round repeatability, {<>,<*s*_*r*_>}, between-participant precision, {<>,<*s*_L_>}, and reproducibility {<>,<*s*_*R*_>} functions displayed in Figure 3 provide one definition of the expected measurement precision of signals from one microarray platform, processed with particular software, obtained by a given group of laboratories at a particular point in time. Comparable precision characteristics for other data preprocessing approaches can be estimated by reanalysis of these data. Evaluating the expected characteristics of other platforms will require new studies, with different samples, but comparable functions can be defined given a similar experimental design and sufficient participants of comparable experience. Whether it will be possible to generalize results among related microarray platforms or across data analysis systems is yet to be assessed.

### Monitoring performance changes over time

**Figure 4:**
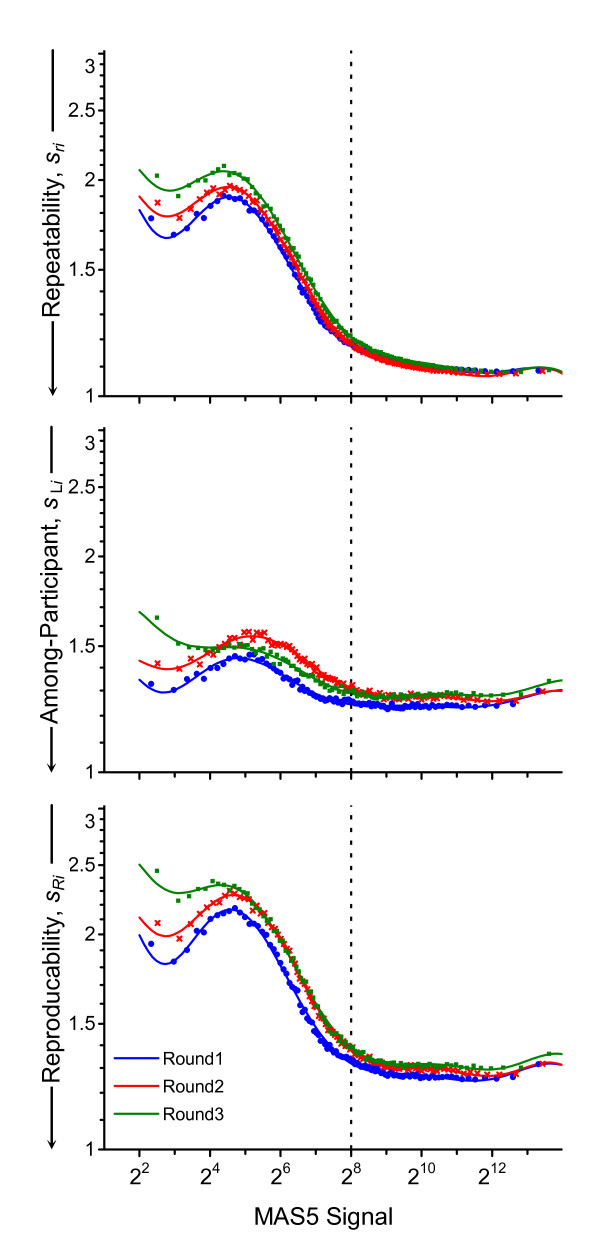
**Change in precision over time**. These panels redisplay the precision functions shown in the right-side panels of Figure 2 but grouped by the type of estimate rather than by round to facilitate quantitative comparison of change with time. The dotted vertical line denotes the median signal level, 2^8^.

The present data are, however, sufficient to evaluate whether the precision characteristics of the particular platform and signal analysis method are stable over time. The discrete within-round {<>,<*s*_*ri*_>} repeatabilities, {<>,<*s*_L*i*_>} between-participant precisions, and {<>,<*s*_*Ri*_>} reproducibilities that are displayed by each round in Figure 2 are redisplayed in Figure 4 by each precision component. The forms of the functions are very similar over the three studies. Curiously, while the level of within-participant repeatability shows little or no trend, the between-participant precision at signal levels above the median (2^8^) appears to have degraded somewhat with time. The invariant repeatability argues against any significant change in the quality of the RNA pools; this plus the distribution of microarrays from multiple lots in Round 1 but single lots in Rounds 2 and 3 argue against significant between-array variability. Thus the small increase in between-participant variability may reflect the changes in the number of participants and the processing protocols that they used; it may also indicate that somewhat less experienced analysts were involved in the later rounds.

### Comparing participant precisions

While simple contrasts of repeatability, *s*_*r*_, and between-participant precision, *s*_L_, (see Methods) enables identification of individual probesets that differ systematically among participants, the variation among the {<>,<*s*(*x*_*ij*_)>} functions in Figure 2 suggests that the systematic differences among the participants are not confined to just a relatively few measurement processes. Comparison of the within-participant repeatability, {<>,<*s*_*rj*_>}, and among-round precision, {<>,<*s*_W*j*_>}, functions for each participant helps identify the nature of long-term changes.

**Figure 5:**
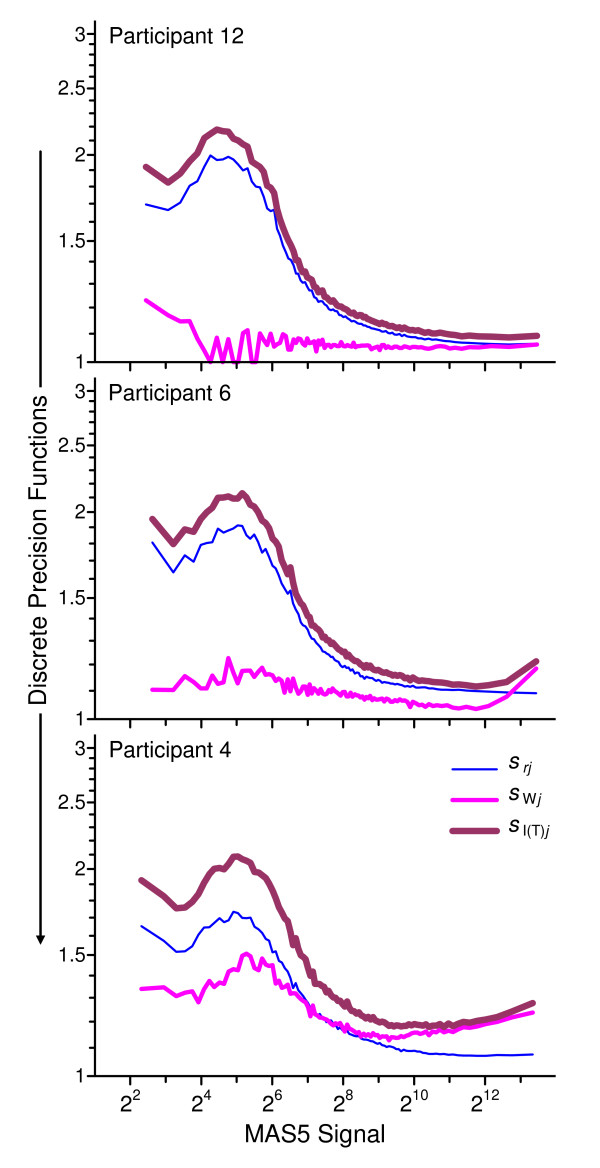
**Example discrete precision functions characteristic of the participant**. These panels display exemplar within-participant repeatability {<>,<*s*_*rj*_>} (thinnest line), among-round precision {<>,<*s*_W*j*_>}, and intermediate precision over time {<>,<*s*_I(T)*j*_>}(thickest line) functions for three participants: 12, 6, and 4.

Figure 5 displays these within-participant discrete functions for three exemplar measurement systems. For participant 12 (top panel), *s*_*r*12 _is quite good at the highest signal levels and *s*_W12 _is both quite good and roughly constant for all levels; this indicates good short-term and excellent long-term control of the measurement process. For participant 6 (middle panel), *s*_*r*6 _is somewhat less good then *s*_*r*12 _with large signals, and while *s*_W6 _is excellent for moderately high signals it is systematically less good at both low and very high levels; this suggests somewhat poorer short-term repeatability and long-term changes in the measurement process that impact high and low signals more than those at the mid-levels. We speculate that these changes may be related to scanner performance, influencing both background noise and signal saturation [23]. For participant 4 (bottom panel), *s*_*r*4 _is generally as good to somewhat better than for *s*_*r*12 _while *s*_W4 _is considerably poorer for all signal levels; this suggests excellent short-term control but significant differences in the (probably pre-scanner) measurement protocol over the course of the three rounds.

**Figure 6:**
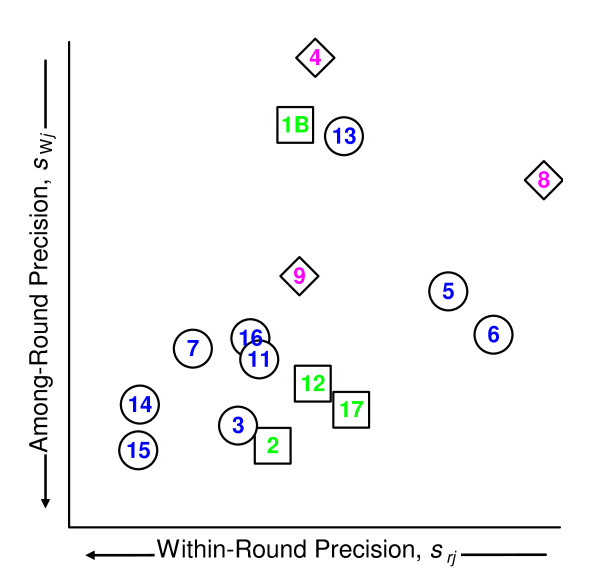
**Comparison of median within- and between-round participant precision**. This scatterplot displays the median among-round precision, *s*_W*j*_, as a function of the median within-participant repeatability, *s*_*rj*_, for the 16 measurement systems used in at least two rounds. The open circles denote protocol group "A" measurement systems, open squares denote group "B" systems, and open diamonds denote group "C" systems.

While multivariate approaches could be used to evaluate and display the relative participant performance based on continuous versions of the short-term and long-term precision functions, the basic structure can be readily visualized from the median value of the discrete functions. Figure 6 displays the median within-round repeatability, *s*_*rj*_, and median among-round precision,*s*_W*j*_. To avoid estimation artifacts, the medians are evaluated over signal levels from 2^8 ^to 2^12^. With three exceptions, between-round precision is limited by within-round performance. The excess between-round variability for measurement systems 1B, 4, and 13 likely results from undocumented changes to the systems. The generally poorer precision for systems in protocol group "C" suggests that one or more factor in this group is not well controlled.

The structure visualized in Figure 6 is congruent with the behavior of the data for the exemplar probeset AFFX_Rat_Hexokinase_5_at discussed in Methods. The association of abstract trends with the behavior of a particular probeset in one sample may facilitate identifying root-causes. The analysis and display tools developed for traditional measurands thus can inform both the development and the interpretation of tools for interlaboratory studies of microarrays.

## Conclusion

The established metrological framework for characterizing precision can be applied to results from microarray interlaboratory studies, enabling precision characteristics of microarray results to be expressed in a way that permits comparison to those of other measurement processes. The design of the Expression Analysis Pilot Proficiency Test facilitated assessment of the nested precision metrics of repeatability, intermediate precision over time, and reproducibility – all critical figures of merit of any analytical method. Such studies and figures of merit are essential tools for objective, quantitative performance assessment of individual laboratories, the population of laboratories, and microarray platforms. We believe that continuous precision functions will prove a vital tool for characterizing and comparing measurement platforms and data processing algorithms.

The tools described here for the simplest of microarray signals, are the foundation for further work addressing precision measures for differential expression and differentially expressed gene lists. Figures of merit for these composite signals will support objective performance assessment of the measures behind the biological inference, the reason for performing the measurements in the first place.

## Methods

### Study design

Expression Analysis, Inc. coordinated three rounds of the Expression Analysis Pilot Proficiency Test in 2004. The first (Round 1) was completed in June, the second (Round 2) in September, and the third (Round 3) in December. All data from these studies are available from ArrayExpress, accession number E-MEXP-1568 . The following summarizes the organizational elements pertinent to our analysis.

#### Participants

Eighteen laboratories participated in at least one of the three rounds, with one laboratory changing its measurement protocol between Round 1 and Round 2. The codes and participation history for the 19 different measurement systems (unique combinations of participant and measurement protocols) are summarized in Table 1. Nine of the measurement systems were used in all three rounds.

**Table 1:** Study design, participation, and protocol groups

		Protocol Groups^a^			
			
	Code	Round 1 6/2004	Round 2 8/2004	Round 3 12/2004	#
					
	1A	A			1
	1B		B	B	2
	2		B	B	2
	3		A	A	2
	4	C	C	C	3
	5	A	A	A	3
	6	A	A	A	3
	7	A	A	A	3
	8	C	C	C	3
	9	C	C	C	3
	10	Y			1
	11	A	A		2
	12	B	B	B	3
	13	A	A		2
	14	A	A	A	3
	15		A	A	2
	16	A	A	A	3
	17		B	B	2
	18			Z	1
					
Total	19	13	16	15	44

a) Four measurement protocol groups, "A" to "C", were identified on the basis of common suppliers of enzymes, purification kits, instrumentation and similar operating conditions. Two participants used unique protocols that are coded "Y" and "Z".

#### Samples

Each participant in each study analyzed three aliquots of two mixed-tissue RNA pools, Mix 1 and Mix 2. The two mixtures were prepared at Expression Analysis by combining different amounts of RNA from rat brain, kidney, liver, and testicle tissues following the design developed by Dr. Karol Thompson and her FDA colleagues [24]. The mixtures were prepared to have total RNA concentrations of 1 μg/μL. Each of the six samples was tagged with unique combinations of five polyadenylated (polyA+) bacterial gene transcripts. The three samples of each mixture were otherwise nominally identical in composition. The sample materials were stored at -80°C by Expression Analysis until shipment and by the participants after receipt. All samples were shipped on dry ice via an overnight delivery service.

#### Microarrays

Each participant received six Rat230 2.0 GeneChips (Affymetrix, Inc, Santa Clara California, USA) for use in each study, one microarray to be used for each RNA sample. Sixteen arrays were replaced because of various participant-recognized technical problems. To study the influence of lot-to-lot variation, four lots of these arrays were used in Round 1. A single array lot was used in Round 2 and a different single lot in Round 3. The GeneChips are 11-μm format. They were annotated to contain 31099 unique probesets, 27 of which were designed to respond to the polyA+ bacterial control transcripts and 18 to respond to hybridization control transcripts. The remaining 31054 probesets were designed to respond to rat RNA.

#### Measurement protocols

Each participant prepared biotin-labeled cRNA targets from each sample and hybridized the cRNA to the rat microarrays using his/her own labeling and hybridization reagents. Each participant followed his/her own standard measurement protocol, with the following restrictions: 1) it was strongly recommended that 10 μg of the RNA sample be used to prepare each target, 2) the biotinylated cRNA was to be prepared using a single round of cDNA synthesis via the Eberwine protocol [25], 3) 20 μg of the fragmented, biotinylated cRNA was to be used with 300 μL of hybridization cocktail including the oligo and eukaryotic controls, and 4) 200 μL of the hybridization cocktail was to be hybridized to each microarray. Three "protocol groups" (coded as "A", "B", and "C") were identified on the basis of the participants' choice of operating conditions and source of enzymes, purification kits, and instrumentation; only two of the 19 measurement systems used during the study were sufficiently different to be considered unique. Table 1 summarizes the use of the different protocols by participant.

#### Signal estimation and processing

Each participant measured the microarrays following their standard protocols using Affymetrix GeneChip Scanner 3000 instruments with high-resolution software. The resulting "CEL" files for each microarray were sent to Expression Analysis for data processing and evaluation where probeset signal values were evaluated using MAS5 [17]. At NIST, the 55 polyA+ and hybridization controls were deleted from all data sets and the remaining 31054 rat RNA signals were log_2 _transformed and centered to have a median log_2_(signal) of 8 (ie, a median signal of 2^8 ^= 256.) This centering value was selected as the integral power of 2 closest to the median of the raw MAS5 signals in all data sets.

#### Data sets

Forty-four sets of data for the six samples were generated over the course of the three rounds. Each data set consists of 31054 probeset signals for three replicate samples of Mix 1 and Mix 2. Table 1 lists the number of data sets returned in each study.

### Classical precision metrics for single studies

Let *P *represent some one particular measurement process, *X *the results generated by that process, and *x *a particular single result. For the data considered here, *P *is the log_2_-transformed MAS5 evaluation of a particular probeset, *X *the set of log_2_-transformed MAS5 results for that probeset in all arrays studied, and *x *the log_2_-transformed MAS5 result for a particular array.

A variety of linear models have been employed to characterize measurement processes from various types of repeated measurements [26]. While complicated models are appropriate when stationary effects (biases) are expected and of interest, given the among-round variation in participants and their measurement protocols, we begin with the general ISO 5725 model. Rather than relying on strong assumptions about the structure of the variance, this simple model is designed to have the general applicability needed for a standard approach. It asserts that each *x *can be expressed as the sum of three components [12]:

(1)

where  is either the true value or more typically a consensus estimate of the quantity being measured, *B *is the systematic difference (bias) between  and the expected result for the given participant's implementation of *P *(as estimated from replicate measurements), and *ε *is the random difference between the expectation for the implementation (ie,  + *B*) and the given value. For a single material evaluated in a single study (that is, at some given point in time), the *ε *are assumed to follow a random distribution centered on zero with a standard deviation associated with the (metrological) repeatability precision characteristic of *P*. Likewise, the variability of the *B *among all participants is assumed to follow a random distribution centered on zero with a standard deviation associated with the (metrological) reproducibility precision of *P*. These terms are described more fully in the *Results Section*; we here detail a standard approach to their estimation.

Let *x*_*ijk *_represent the *k*^th ^of *N*_*m *_replicate measurements of *P *reported by the *j*^th ^participant in the *i*^th ^study. The standard deviation of the replicates, *s*(*x*_*ij*_), estimates the random variability of *P *as implemented by the *j*^th ^participant in the *i*^th ^study:

(2)

where  is the mean of the replicates. Assuming that the variance magnitude is roughly similar for all participants (as is the case, see Figure 2), the expected random variability of *P *common to all participants (*i.e*., its repeatability precision) in the *i*^th ^study, *s*_*ri*_, can be estimated by combining the individual *s*(*x*_*ij*_) over all *N*_*pi *_participants. While the general formulae detailed in [12] describe variable numbers of replicate measurements for different participants, for these data the same numbers of replicates were reported by every participant in every round. The appropriate formula for pooling variance in this circumstance is:

(3)

The *s*_*ri *_can be interpreted as the "average" standard deviation expected for technical replicate measurements made by a typical laboratory, where "typical" is defined by the population of actual participants.

The between-participant precision for the *i*^th ^study, *s*_L*i*_, is estimated from the standard deviation of the estimated participant-specific biases:

(4)

where *max*() is the "take the maximum value of the arguments" function and  is the mean of the participant mean values. The  term estimates for the repeatability contribution to the variance of the biases. With atypically noisy replicate measurements, the corrected bias variance is defined as zero – allocating the observed variance to the least-complex source. The *s*_L*i *_estimates the extent of agreement among the various implementations of *P *used by the participants. Ideally, all participants will observe the same mean value for *X *and the value for *s*_L*i *_will be near zero; in practice, studies involving more than one measurement protocol often (by conscious study or from hard experience) discover *s*_L*i *_to be several times larger than *s*_*ri*_.

The reproducibility of *P *during the *i*^th ^study, *s*_*Ri*_, is estimated by combining the *s*_*ri *_and *s*_L*i *_variance components:

(5)

The *s*_*Ri *_combines all of the factors influencing *P *at the time the study was performed; the implementation of *P *in a typical laboratory is expected to yield results that agree with results of other such users within confidence limits appropriate to a normal distribution having mean  and standard deviation *s*_*Ri*_.

**Figure 7:**
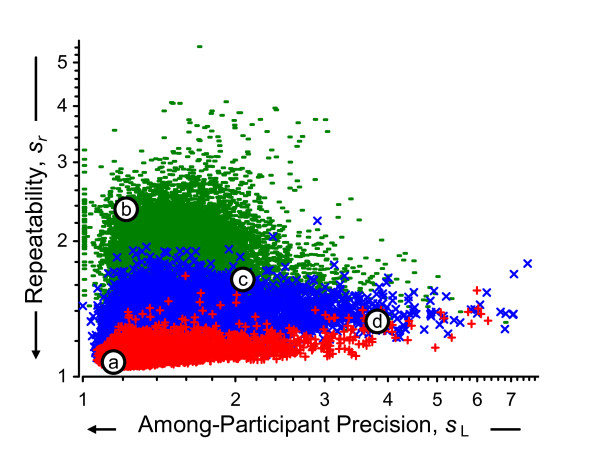
**Within-round between-participant and repeatability precisions**. This scatterplot displays 62108 repeatabilities for Round 1 data, *s*_*r*1_, as a function of the between-participant precisions, *s*_L1_, and the average log_2 _signal, , for the Mix 1 and Mix 2 technical replicates. The one-third of probesets with the smallest  are denoted green "-", the middle third are denoted blue "×", and one-third with the largest  are denoted red "+". The labeled open circles denote the {*s*_L1_, *s*_*r*1_} location of four exemplar probesets. The precisions are expressed as multiplicative standard deviations: a value of 1 indicates that the values are within a factor of 1 of their mean (i.e., they are identical) whereas a value of 5 indicates a 5-fold spread about their mean.

The notation used in the above calculations is summarized in Additional file 1. Additional file 2 lists the data and results for the above calculations for one exemplar probeset. Figure 7 displays the relationships among the above estimates for all 31054 probesets in Round 1. While repeatability magnitude is related to signal level, there is considerable variety among the magnitude of the between-participant precision regardless of level. A considerable number of the *s*_L1 _are plotted along the left-hand margin (*s*_L1 _= 2^0 ^= 1), a consequence of estimating variance with a relatively small number of replicate measurements.

**Figure 8:**
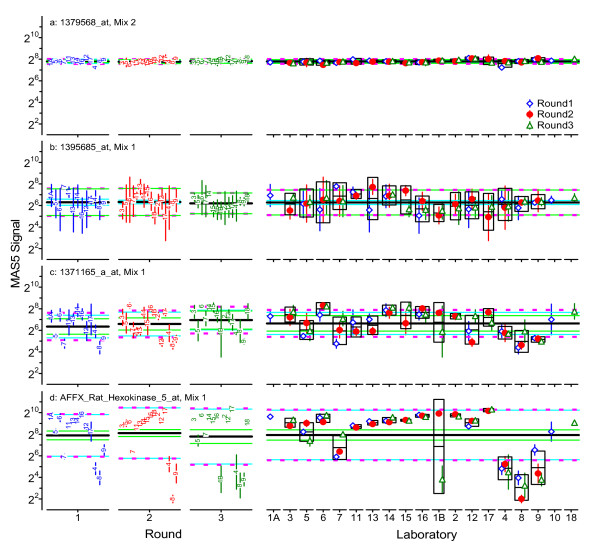
**Example graphical analysis of within-round precisions for individual probesets**. This "dot-and-bar" chart provides a graphical summary of the participant averages, , and standard deviations, *s*(*x*_1*i*_), for the four probesets labeled in Figure 7. The within-round averages, , are represented as a thick black line; the repeatabilities, *s*_*r*1_, as solid green lines one precision unit on either side of average; the between-participant precision, s_L1_, as solid light-blue lines; and the reproducibilities, *s*_*R*1_, as dashed magenta lines. Participant codes are ordered by protocol group: Group A {1A,5,6,7,11,13,14,16}, Group B {12,17}, and Group C {4,8,9}.

Additional file 3 summarizes the results of the above calculations for four exemplar probesets in all three rounds. Figure 8 displays these results in a "dot-and-bar" format commonly used with interlaboratory studies. These four probesets were selected as typical of the observed range of the {*s*_L1_, *s*_*r*1_} pairs displayed in Figure 7, where the {*s*_L1_, *s*_*r*1_} locations are marked with open circles labeled *a *to *d*. Exemplar 1, probeset 1379568_at of Mix 2, has very small *s*_L1 _and *s*_*r*1_; this represents results that are about the same for all participants, for all replicate samples. Exemplar 2, 1395685_at of Mix 1, has small *s*_L1 _but large *s*_*r*1_; this represents results with considerable technical variability but with averages that are about the same for all participants participants. Exemplar 3, 1371165_a_at of Mix 1, has moderate *s*_L1 _and *s*_*r*1_; this represents modest variability with some systematic differences among the participants. Exemplar 4, AFFX_Rat_Hexokinase_5_at of Mix 1, has large *s*_L1 _but relatively small *s*_*r*1_; this represents results with considerable and quite consistent systematic differences among the participants.

### Classical precision metrics for multiple studies

When results for two or more qualitatively similar interlaboratory studies are available, the individually short-term study-specific estimates can be used to define the long-term performance characteristics of the measurement process, *P*, with great confidence. It may also be possible to explore the temporal stability of participant-specific systematic bias, *B*, and random variability, *ε*. Indeed, an explicit goal of many interlaboratory studies is to help participants identify and minimize sources of systematic difference in their individual implementations of *P *and to establish tighter statistical control over its random influences [27]. Changes in individual performance will manifest may manifest as changes in the study-specific repeatability and reproducibility estimates [28].

Given *N*_*s *_studies that evaluate identical samples, laboratory-specific repeatabilities can be estimated for all participants that use nominally identical implementations of *P *in at least two of the studies. For the *j*^th ^such laboratory, *s*_*rj *_is estimated by combining the simple standard deviations, *s*(*x*_*ij*_), over the *N*_*sj *_studies in which they participated. Since here the same number of replicates were evaluated in each round, the general pooling formula again simplifies to:

(6)

In a manner analogous to the between-participant precision described above, laboratory-specific estimates can be obtained for the extent of agreement among results they obtain over relatively long periods of time. The among-round precision for the *j*^th ^participant, *s*_W*j*_, is calculated:

(7)

where  is the mean of the mean values for the particular laboratory over all of the studies in which they participated. The intermediate precision over time for the participant, *s*_I(T)*j *_[13], is the combination of *s*_*rj *_and *s*_W*j*_:

(8)

Additional file 4 summarizes these long-term within-participant precision calculations for the four exemplar probesets.

The long-term expected value for *X*, , and the total number of sets of *X *values reported by all participants in all of the studies, *N*_*t*_, can be calculated across the *N*_*p *_participants or across the *N*_*s *_studies:

(9)

The expected long-term repeatability, *s*_*r*_, is directly calculated from the participation-weighted average of the laboratory-specific repeatability variances; however, the same value is obtained by pooling the study-specific repeatability estimates:

(10)

Similarly, the expected long-term between-laboratory precision, *s*_L_, is calculated from the study size-weighted average of the between-laboratory precision variances:

(11)

The expected long-term reproducibility of the measurement process, *s*_*R*_, can be calculated from the study size-weighted average of the study-specific reproducibility variances or by combining *s*_*r *_and *s*_L_:

(12)

**Figure 9:**
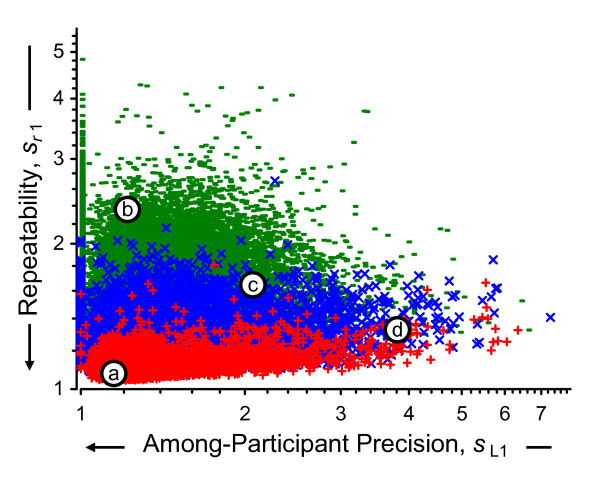
**Between-round between-participant and repeatability precisions**. This scatterplot is identical in format to Figure 7, only displaying the overall between-participant precision and repeatability estimates pairs, {*s*_L_, *s*_*r*_} rather than just those of Round 1.

**Figure 10:**
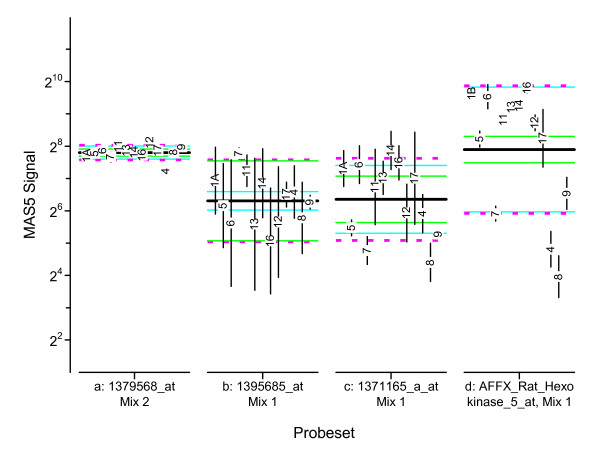
**Example graphical analysis of between-round precisions for individual probesets**. The left section of each panel displays dot-and-bar charts for the three rounds in the format used in Figure 8. The right section displays the same participant average and standard deviation dot-and-bar data, but grouped by participant rather than round. The mid-line of the box around the data for each participant represents that participant's average, ; the lower and upper edges of the box denote the intermediate precision over time, *s*_I(T)*j*_. The grand average, , is represented as a think black line; the repeatability, *s*_*r*_, as solid green lines; the between-participant precision, s_L_, as solid light-blue lines; and the reproducibility, *s*_*R*_, as dashed magenta lines. Participant codes are ordered by protocol group: Group A {1A,3,5,6,7,11,13,14,15,16}, Group B {1B,2,12,17}, Group C {4,8,9}, Y {10}, and Z {18}.

Figure 9 displays the long-term repeatabilities and between-laboratory precisions characteristic of the microarray platform for all of the 31054 probesets. The relationship of the precision estimates to the signal level is not changed from Figure 7, although the increased number of data used in the estimates can be seen in the much reduced number of probesets with *s*_L _= 2^0 ^= 1. Note that the locations of the exemplar probesets relative to {*s*_L_, *s*_*r*_} are unchanged after three rounds. Figure 10 displays all of the above precision estimates for the four exemplar probesets.

### "Outliers"

It is often necessary to identify and remove grossly aberrant values and/or use robust statistical estimation techniques to enable sensible summarization of a given data set. Other than the arrays that were replaced because of participant-recognized technical problems, no "outlier arrays" (in the sense of the majority of MAS5 values for one of the three replicate arrays being quite different from those of its siblings) were identified in the final data. Robust methods, including those advocated in ISO 5725-5 [29], were evaluated and found to yield estimates very similar to those described above.

## Authors' contributions

DLD participated in the development of the precision estimators, coded the calculations, and drafted the manuscript. WDJ and LHR designed the interlaboratory studies, collected and processed the resulting data, and advised on their interpretation. MS participated in the development of the precision estimators and drafting the manuscript. All authors read and approved the final manuscript.

## Supplementary Material

Additional file 1 **Summary of notation**. Summarizes the notation used with the various calculations detailed in Methods. Click here for file

Additional file 2 **Data and precision calculations for RNA Mix 1, exemplar probeset AFFX_Rat_Hexokinase_5_at**. Provides the data and detailed results of the various calculations for one of the exemplar probesets described in Methods. Click here for file

Additional file 3 **Summary of method-related precision estimates for four exemplar probesets**. Provides numeric values for the various method-related precision estimates for the four exemplar probesets described in Methods. Click here for file

Additional file 4 **Summary of participant-related precision estimates for four exemplar probesets**. Provides numeric values for the various participant-related precision estimates for four exemplar probesets described in Methods. Click here for file
