# Supplementary material for: Learning from microarray interlaboratory studies: measures of precision for gene expression
Source: BMC Genomics. 2009 Apr 8;10:153. doi: 10.1186/1471-2164-10-153 (PMC2679054; doi:10.1186/1471-2164-10-153)
Supplement: Additional file 1 — Summary of notation. Summarizes the notation used with the various calculations detailed in Methods. [file 1471-2164-10-153-S1.pdf]

Additional file 1: Summary of Notation

i .... index of the “rounds” of the interlaboratory comparison program
j .... index of the participants in each round
k................................ index of the replicate measurements made by each participant in each round
N_m............................. number of replicate measurements; N_m is always 3 for the data of this study
N_p .... total number of participants
N_{pi}............................. number of participants in the ith round
N_s.............................. total number of rounds
N_{sj} .... number of rounds the jth participant participated in
N_t.............................. total number of sets of replicate measurements over all rounds and participants
$s(x_{ij})$.......................... participant standard deviation for one set of replicate X measurements
s_{L} .... between-participant precision of the measurement process
$s_{\mathrm{L}i}$.............................. between-participant precision in the ith round
s_r .... repeatability of the measurement process
s_{ri} .... repeatability in the ith round
s_{rj} .... repeatability of the jth participant
s_R .... reproducibility of the measurement process
s_{Ri}.............................. reproducibility in the ith round
$s_{\mathrm{I(T)}j}$ .... intermediate precision over time for the jth participant
$s_{\mathrm{W}j}$............................. among-round precision for the jth participant
X............................... a particular expression measure; here, $\log_2$(MAS5)
x_{ijk} .... k^{th} X measurement by j^{th} participant in i^{th} round
$\bar{x}_{ij}$ .... average of replicate measurements for the jth participant in i^{th} round
$\bar{\bar{x}}_i$ .... average of averages in the ith round
$\bar{\bar{x}}_j$ .... average of averages for the jth participant over all rounds
$\bar{\bar{\bar{x}}}$ .... grand average of averages over all rounds and participants
$<\cdot>$............................ mean or median of a list of independent values
$\{\cdot,\cdot\}$........................... set of two or more identically ordered lists
