# Supplementary material for: Learning from microarray interlaboratory studies: measures of precision for gene expression
Source: BMC Genomics. 2009 Apr 8;10:153. doi: 10.1186/1471-2164-10-153 (PMC2679054; doi:10.1186/1471-2164-10-153)
Supplement: Additional file 2 — Data and precision calculations for RNA Mix 1, exemplar probeset AFFX_Rat_Hexokinase_5_at. Provides the data and detailed results of the various calculations for one of the exemplar probesets described in Methods. [file 1471-2164-10-153-S2.pdf]

Additional file 2: Data and precision calculations for RNA Mix 1, exemplar probeset AFFX_Rat_Hexokinase_5_at.
All estimates are for $\log_2$-transformed MAS5 signals.

	Round 1					Round 2					Round 3					Between-Round				
Code	x_{1jA}	x_{1jB}	x_{1jC}	$\bar{x}_{1j}$	s_{1j}	x_{2jA}	x_{2jB}	x_{2jC}	$\bar{x}_{2j}$	s_{2j}	x_{3jA}	x_{3jB}	x_{3jC}	$\bar{x}_{3j}$	s_{3j}	N_{sj}	$\bar{\bar{x}}_j$	s_{rj}	s_{Wj}	$s_{I(T)j}$
1A	9.714	9.500	9.687	9.634	0.117											1	9.634	0.117	*na*	*na*
1B						9.829	9.965	9.946	9.913	0.073	5.060	2.591	3.822	3.824	1.234	2	6.869	0.874	4.276	4.364
2						9.933	9.703	9.893	9.843	0.123	9.632	9.699	9.662	9.664	0.034	2	9.754	0.090	0.115	0.146
3						8.772	8.703	8.860	8.778	0.078	9.235	9.489	9.349	9.358	0.127	2	9.068	0.106	0.405	0.419
4	4.585	4.408	5.438	4.810	0.551	5.641	4.416	5.649	5.235	0.710	5.516	5.334	2.658	4.503	1.600	3	4.849	1.060	0.000	1.060
5	8.434	8.279	7.950	8.221	0.247	9.216	9.187	8.665	9.023	0.310	7.454	6.881	7.903	7.413	0.512	3	8.219	0.374	0.776	0.861
6	9.806	9.687	9.105	9.533	0.375	9.263	8.944	9.202	9.136	0.169	9.784	9.756	9.790	9.777	0.018	3	9.482	0.238	0.293	0.377
7	5.661	6.118	5.956	5.912	0.232	6.422	6.366	6.385	6.391	0.029	8.058	7.845	8.096	8.000	0.135	3	6.768	0.156	1.090	1.101
8	3.782	3.422	4.678	3.961	0.647	2.098	2.300	1.598	1.999	0.361	4.523	2.979	2.270	3.257	1.152	3	3.072	0.791	0.883	1.185
9	5.990	6.948	6.685	6.541	0.495	4.615	5.090	3.390	4.365	0.877	4.005	3.131	4.295	3.810	0.606	3	4.906	0.679	1.389	1.546
10	9.234	8.007	7.497	8.246	0.893											1	8.246	0.893	*na*	*na*
11	8.898	8.664	8.886	8.816	0.132	8.465	8.734	8.656	8.619	0.138						2	8.717	0.135	0.116	0.178
12	8.977	8.494	8.690	8.720	0.243	9.342	9.342	9.016	9.233	0.188	9.275	9.291	9.298	9.288	0.012	3	9.081	0.178	0.296	0.345
13	9.333	9.186	9.108	9.209	0.114	8.947	8.830	9.041	8.940	0.106						2	9.074	0.110	0.180	0.211
14	9.301	9.343	9.101	9.248	0.129	9.155	9.146	9.053	9.118	0.056	9.607	9.574	9.556	9.579	0.026	3	9.315	0.083	0.233	0.247
15						9.310	9.330	9.292	9.311	0.019	9.138	9.373	9.341	9.284	0.127	2	9.297	0.091	0.000	0.091
16	9.959	9.644	9.786	9.796	0.158	9.765	9.817	9.555	9.712	0.139	9.631	9.717	9.546	9.631	0.085	3	9.713	0.131	0.034	0.135
17						10.080	10.140	10.271	10.164	0.098	10.445	10.362	10.149	10.319	0.153	2	10.241	0.128	0.081	0.152
18											9.192	9.085	8.944	9.074	0.124	1	9.074	0.124	*na*	*na*
			N_{p1}	13				N_{p2}	16				N_{p3}	15		44			N_t	
			$\bar{\bar{x}}_1$	7.896				$\bar{\bar{x}}_2$	8.111				$\bar{\bar{x}}_3$	7.785			7.937		$\bar{\bar{x}}$	
			s_{r1}		0.408			s_{r2}		0.321			s_{r3}		0.640			0.476	s_r	
			s_{L1}	1.931				s_{L2}	2.340				s_{L3}	2.539				2.300	s_L	
			s_{R1}	1.973				s_{R2}	2.362				s_{R3}	2.618				2.348	s_R	
