# Supplementary material for: Learning from microarray interlaboratory studies: measures of precision for gene expression
Source: BMC Genomics. 2009 Apr 8;10:153. doi: 10.1186/1471-2164-10-153 (PMC2679054; doi:10.1186/1471-2164-10-153)
Supplement: Additional file 3 — Summary of method-related precision estimates for four exemplar probesets. Provides numeric values for the various method-related precision estimates for the four exemplar probesets described in Methods. [file 1471-2164-10-153-S3.pdf]

Additional file 3: Summary of method-related precision estimates for four exemplar probesets. All estimates are for $\log_2$-transformed MAS5 signals.

Statistic	a) Mix2 G2_1379568_at	b) Mix1 G1_1395685_at	c) Mix1 G1_1371165_a_at	d) Mix1 AFFX_Rat_Hexokinase_5_at
N_{p1}	13	13	13	13
$\bar{\bar{x}}_1$	7.801	6.309	6.355	7.896
s_{r1}	0.108	1.236	0.717	0.408
s_{L1}	0.202	0.285	1.049	1.931
s_{R1}	0.228	1.269	1.271	1.973
N_{p2}	16	16	16	16
$\bar{\bar{x}}_2$	7.780	6.328	6.590	8.111
s_{r2}	0.182	1.255	0.567	0.321
s_{L2}	0.129	0.172	1.102	2.340
s_{R2}	0.223	1.267	1.239	2.362
N_{p3}	15	15	15	15
$\bar{\bar{x}}_3$	7.817	6.202	6.956	7.785
s_{r3}	0.189	0.957	0.844	0.640
s_{L3}	0.071	0.000	0.910	2.539
s_{R3}	0.202	0.957	1.241	2.618
N_t	44	44	44	44
$\bar{\bar{x}}$	7.799	6.279	6.645	7.937
s_r	0.166	1.156	0.715	0.476
s_L	0.140	0.186	1.024	2.300
s_R	0.218	1.171	1.249	2.348
