# Supplementary material for: Learning from microarray interlaboratory studies: measures of precision for gene expression
Source: BMC Genomics. 2009 Apr 8;10:153. doi: 10.1186/1471-2164-10-153 (PMC2679054; doi:10.1186/1471-2164-10-153)
Supplement: Additional file 4 — Summary of participant-related precision estimates for four exemplar probesets. Provides numeric values for the various participant-related precision estimates for four exemplar probesets described in Methods. [file 1471-2164-10-153-S4.pdf]

Additional file 4: Summary of participant-related precision estimates for four exemplar probesets.
All estimates are for $\log_2$-transformed MAS5 signals.

		a) Mix2 G2_1379568_at				b) Mix1 G1_1395685_at				c) Mix1 G1_1371165_a_at				d) Mix1 AFFX_Rat_Hexokinase_5_at			
Code	Ns_j	$\bar{\bar{x}}_j$	s_{rj}	s_{Wj}	$s_{I(T)j}$	$\bar{\bar{x}}_j$	s_{rj}	s_{Wj}	$s_{I(T)j}$	$\bar{\bar{x}}_j$	s_{rj}	s_{Wj}	$s_{I(T)j}$	$\bar{\bar{x}}_j$	s_{rj}	s_{Wj}	$s_{I(T)j}$
1A	1	7.710	0.110	*na*	*na*	6.933	1.033	*na*	*na*	7.308	0.555	*na*	*na*	9.634	0.117	*na*	*na*
1B	2	7.881	0.120	0.000	4.364	5.307	0.799	0.000	4.364	6.749	1.679	0.745	4.364	6.869	0.874	4.276	4.364
2	2	7.885	0.089	0.090	0.146	5.942	1.265	0.000	0.146	7.309	0.247	0.000	0.146	9.754	0.090	0.115	0.146
3	2	7.654	0.047	0.000	0.419	6.144	0.620	0.780	0.419	7.466	0.688	0.000	0.419	9.068	0.106	0.405	0.419
4	3	7.596	0.109	0.311	1.060	6.108	1.490	0.000	1.060	5.787	0.584	0.000	1.060	4.849	1.060	0.000	1.060
5	3	7.753	0.296	0.000	0.861	6.236	1.299	0.000	0.861	6.035	0.774	0.407	0.861	8.219	0.374	0.776	0.861
6	3	7.682	0.159	0.171	0.377	6.294	1.896	0.000	0.377	7.991	0.382	0.436	0.377	9.482	0.238	0.293	0.377
7	3	7.733	0.128	0.000	1.101	6.867	1.178	0.393	1.101	5.960	0.627	1.104	1.101	6.768	0.156	1.090	1.101
8	3	7.788	0.189	0.000	1.185	6.149	0.688	0.000	1.185	4.982	0.734	0.701	1.185	3.072	0.791	0.883	1.185
9	3	7.949	0.134	0.140	1.546	6.359	0.653	0.000	1.546	5.159	0.244	0.071	1.546	4.906	0.679	1.389	1.546
10	1	7.865	0.119	*na*	*na*	6.503	0.384	*na*	*na*	7.007	1.428	*na*	*na*	8.246	0.893	*na*	*na*
11	2	7.816	0.115	0.239	0.178	7.073	0.419	0.000	0.178	6.312	0.867	0.331	0.178	8.717	0.135	0.116	0.178
12	3	8.092	0.114	0.000	0.345	6.024	1.249	0.000	0.345	6.055	0.655	1.162	0.345	9.081	0.178	0.296	0.345
13	2	7.822	0.114	0.000	0.211	6.652	1.526	1.225	0.211	6.474	0.447	0.727	0.211	9.074	0.110	0.180	0.211
14	3	7.805	0.134	0.000	0.247	6.937	0.883	0.000	0.247	7.873	0.464	0.000	0.247	9.315	0.083	0.233	0.247
15	2	7.678	0.058	0.063	0.091	6.523	0.608	1.153	0.091	7.388	0.820	0.920	0.091	9.297	0.091	0.000	0.091
16	3	7.723	0.211	0.000	0.135	5.692	1.189	0.000	0.135	7.665	0.541	0.023	0.135	9.713	0.131	0.034	0.135
17	2	7.858	0.325	0.113	0.152	5.421	1.707	0.000	0.152	7.202	0.569	0.577	0.152	10.241	0.128	0.081	0.152
18	1	8.040	0.244	*na*	*na*	6.728	0.380	*na*	*na*	7.767	0.722	*na*	*na*	9.074	0.124	*na*	*na*
